# Adamantane Functionalized Poly(2-oxazoline)s with Broadly Tunable LCST-Behavior by Molecular Recognition

**DOI:** 10.3390/polym13030374

**Published:** 2021-01-26

**Authors:** Joachim F. R. Van Guyse, Debaditya Bera, Richard Hoogenboom

**Affiliations:** Supramolecular Chemistry Group, Centre of Macromolecular Chemistry (CMaC), Department of Organic and Macromolecular Chemistry, Ghent University, Krijgslaan 281-S4, B-9000 Ghent, Belgium; joachimvanguyse@gmail.com (J.F.R.V.G.); beradebaditya@gmail.com (D.B.)

**Keywords:** stimuli-responsive polymer, post-polymerization modification, supramolecular association, poly(2-oxazoline)s, cyclodextrin

## Abstract

Smart or adaptive materials often utilize stimuli-responsive polymers, which undergo a phase transition in response to a given stimulus. So far, various stimuli have been used to enable the modulation of drug release profiles, cell-interactive behavior, and optical and mechanical properties. In this respect, molecular recognition is a powerful tool to fine-tune the stimuli-responsive behavior due to its high specificity. Within this contribution, a poly(2-oxazoline) copolymer bearing adamantane side chains was synthesized via triazabicyclodecene-catalyzed amidation of the ester side chains of a poly(2-ethyl-2-oxazoline-*stat*-2-methoxycarbonylpropyl-2-oxazoline) statistical copolymer. Subsequent complexation of the pendant adamantane groups with sub-stoichiometric amounts (0–1 equivalents) of hydroxypropyl β-cyclodextrin or β-cyclodextrin enabled accurate tuning of its lower critical solution temperature (LCST) over an exceptionally wide temperature range, spanning from 30 °C to 56 °C. Furthermore, the sharp thermal transitions display minimal hysteresis, suggesting a reversible phase transition of the complexed polymer chains (i.e., the β-cyclodextrin host collapses together with the polymers) and a minimal influence by the temperature on the supramolecular association. Analysis of the association constant of the polymer with hydroxypropyl β-cyclodextrin via ^1^H NMR spectroscopy suggests that the selection of the macrocyclic host and rational polymer design can have a profound influence on the observed thermal transitions.

## 1. Introduction

The ability of a material to undergo a physical or chemical change in response to a change in its immediate environment, viz. adapt, is generally considered as an attractive feature, whereby clever design and engineering allows one to exploit this behavior to develop next-generation materials [1,2]. For this reason, polymers that respond to various stimuli, such as temperature [3], pH [4], light [5], mechanical stress [6,7], and oxidative and electrical potentials [8,9] have seen increased use in advanced applications such as drug delivery, tissue engineering, sensing applications, and shape memory materials [1,2]. One frequently exploited adaptive behavior is the phase transition of a polymer solution as a response to a change in temperature, whereby a lower critical solution temperature (LCST) behavior entails phase separation as the temperature increases (i.e., an entropy driven phase separation) [10], while upper critical solution temperature (UCST) behavior entails phase separation upon cooling (i.e., an enthalpy-driven phase separation) [11]. These processes and the temperature at which phase separation occurs are highly dependent on the concentration and ionic strength of the solution, as well as the polymer composition [12,13,14]. For LCST behavior in particular, the hydrophilic–hydrophobic balance in the polymer structure can be carefully optimized to tune the LCST behavior, or more specifically, the phase separation temperature or the cloud point temperature (T_cp_) at a given polymer concentration. In this regard, numerous hydrophilic–hydrophobic monomer combinations have been explored for several biocompatible polymer classes, such as poly(acrylamides) [15,16,17], oligoethyleneglycol functional polymers [10,18,19], poly(amino acids), polypeptoids, and poly(2-alkyl-2-oxazoline)s (PAOx) [20,21,22,23,24], and they are subsequently exploited in tissue engineering or drug delivery contexts [25,26,27,28,29,30]. While tuning the LCST behavior by varying the relative ratio of hydrophilic to hydrophobic monomers might seem straightforward, several factors have to be taken into account, viz. the reactivity ratios of the respective monomers and the resulting monomer distribution along the polymer chain, which can complicate the accurate tuning of the T_cp_. Furthermore, the relationship between the monomer feed ratio and the observed T_cp_ is not necessarily linear, as multiple studies have shown complex exponential relationships, even for near-ideal random copolymers [31,32,33,34,35,36,37,38]. Hence, considerable synthetic effort and thorough characterization are required to thoroughly understand and accurately tune the thermoresponsive behavior as a function of polymer structure.

**Figure 1:**
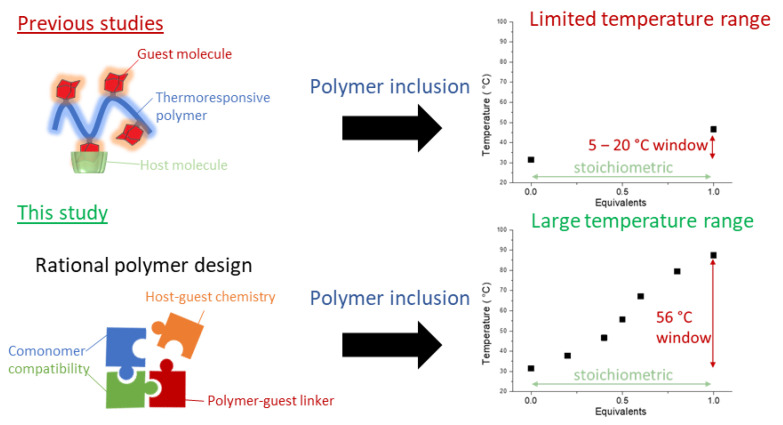
Schematic illustration on the application of rational polymer design through post-polymerization modification in combination with host–guest chemistry (polymer inclusion), presented in this work toward the development of widely tunable lower critical solution temperature (LCST) behavior.

A potential alternative strategy to tune the LCST behavior of a polymer is the utilization of molecular recognition or supramolecular complexation in order to mask the hydrophobic parts of a given polymer, which results in an increase in the T_cp_ [39]. For this purpose, several hydrophilic hosts have been utilized to accommodate a variety of pendant hydrophobic groups on the polymer chains. Most notable is the utilization of various macrocycles, such as cucurbit[7]urils [40], pillararenes [41,42], and cyclobis(paraquat-*p*-phenylene)s [43,44] to modulate the LCST behavior of thermoresponsive polymers through complexation with polymer termini or side chains [40,45,46,47,48,49,50,51]. Among these different hosts, cyclodextrins have been prominently featured for the modulation of thermoresponsive behavior, as they can accommodate a wide variety of hydrophobic guest molecules, have an extensively studied complexation chemistry, and various sizes (α, β, and γ-cyclodextrin) [52] and chemical variants are commercially available or can be readily prepared [53,54]. In addition, cyclodextrins and several of their derivatives are generally recognized as safe, which is ideal for biomedical applications [55,56]. Thus far, they have been utilized to complex polymers with pendant adamantane groups [45,46,47], various aromatic systems [48,49], long or cyclic alkyl chains [40,46,50,51], and even some polymer backbones [51], resulting in the formation of pseudorotaxanes. Despite this structurally diverse pool of potential polymer inclusion complexes that can be formed, the ability of cyclodextrins, and macrocyclic hosts in general, to elicit large shifts in T_cp_ upon complexation is limited. In most cases, a substantial molar excess of the host has to be added to elicit a small shift in the T_cp_, where most systems display a small shift of 5–20 °C in the T_cp_ upon full complexation. The highest reported shift in T_cp_ elicited by a macrocyclic host so far is 30 °C at equimolarity upon complexation of the nonyl side chains of a poly(2-ethyl-2-oxazoline-*ran*-2-nonyl-2-oxazoline) copolymer with a cucurbit[7]uril host [40]. Treatment of the same systems with various cyclodextrins only led to a shift of 10–15 °C at equimolarity [40]. This limited the control over the T_cp_, and the need for the stoichiometric excess of the host strongly limits their advantages over non-supramolecular systems and their applicability in settings where thermoresponsive behavior can be exploited, being mainly limited to sensing applications. In order to increase the attractiveness and applicability of polymer inclusion complexes, efforts should be aimed at designing systems that enable the tuning of the T_cp_ over a wide range, with substoichiometric quantities (0–1 equivalents) of the host as well as systems characterized by high association constants. The latter not only reduces the required quantity of the host necessary to elicit a change in thermoresponsive behavior, but also entails the robustness of the system in biological settings, where a free host is subject to constant removal. Inspired by these challenges, we report the rational design of a poly(2-alkyl-2-oxazoline) (PAOx) copolymer with pendant adamantane groups, which allows straightforward tuning of the T_cp_ over a 30–56 °C range by the addition of substoichiometric quantities (0–1 equivalents) of β-cyclodextrin derivatives, as illustrated in Figure 1. Furthermore, the formed polymer inclusion complexes display sharp thermal transitions upon heating and cooling with minimal hysteresis over the entire temperature range, suggesting a reversible phase transition of the complexed polymer chains (i.e., the β-cyclodextrin host collapses together with the polymers) and a minimal influence of temperature on the supramolecular association. Together, these results demonstrate the potential of polymer inclusion complexes to provide a high degree of tunability and to accurately control the thermoresponsive properties in a simple and straightforward manner.

## 2. Materials and Methods

### 2.1. Materials

The following chemicals were used as received, unless otherwise stated. Barium oxide (BaO, 90%), magnesium sulfate (MgSO_4_, anhydrous, 97%), and 2-chloroethylamine hydrochloride (98%) were purchased from Acros Organics (Geel, Belgium). Sodium methoxide (NaOMe, 95%), thionyl chloride (SOCl_2_, ≥99%), sodium carbonate (Na_2_CO_3_, anhydrous, >99%), piperidine (99%), methyl *p*-toluenesulfonate (MeOTs, 98%), dichloromethane (DCM, ≥99%), diethylether (Et_2_O, >99%), triethylamine (TEA, 99%), (2-hydroxypropyl)-β-cyclodextrin (average M_w_~1540), LiCl (anhydrous, >99%), and 1,5,7-Triazabicyclo[4.4.0]dec-5-ene (TBD, 98%) were purchased from Sigma-Aldrich (Overijse, Belgium). Succinic anhydride (95%) and 1-adamantanemethylamine (98%) were purchased from TCI (Zwijndrecht, Belgium). The 2-Ethyl-2-oxazoline (EtOx) was kindly provided by polymer chemistry innovations (Tuscon, AZ, USA), and was further purified by distilling over BaO and ninhydrin. The β-Cyclodextrin was kindly provided by Wacker Chemie (Munich, Germany). Deuterated water (D_2_O), dimethylsulfoxide (DMSO-*d6*), and chloroform (CDCl_3_) were purchased from Eurisotop (Saint-Aubin, France). The 2-methoxycarbonylpropyl-2-oxazoline (C3MestOx) was synthesized by following a previously reported protocol [57]. The cyclodextrins were dried overnight under vacuum at 50 °C before use. The piperidine was dried over 4 Å molecular sieves before use.

### 2.2. Equipment

The 1D ^1^H spectra were measured on a Bruker Avance Il spectrometer operating at a ^1^H frequency of 400.13 MHz and equipped with a 5 mm PABBO BB− probe. Alternatively, the 1D ^1^H and diffusion ordered spectroscopy (DOSY) spectra were measured on a Bruker Avance Il spectrometer, operating at a ^1^H frequency of 500.13 MHz and equipped with a 5 mm 1H 13C 19F triple resonance observe (TXO) probe. For each sample measurement, the sample temperature was set at 25 °C (and controlled within ± 0.1 °C with a Eurotherm 2000 VT controller), and the chemical shifts were given in parts per million (δ) relative to tetramethylsilane. Size-exclusion chromatography (SEC) was performed on an Agilent 1260-series HPLC system equipped with a 1260 online degasser, a 1260 ISO-pump, a 1260 automatic liquid sampler (ALS), a thermostatted column compartment (TCC) set at 50 °C equipped with two PLgel 5 µm mixed-D columns (7.5 mm × 300 mm) and a precolumn in series, a 1260 diode array detector (DAD), and a 1260 refractive index detector (RID). The used eluent was *N,N*-dimethylacetamide (DMA, HPLC-grade, Sigma-Aldrich) containing 50 mM of LiCl at a flow rate of 0.500 mL/min. The spectra were analyzed using the Agilent Chemstation software with the GPC add-on. The molar mass values and molar mass distribution (i.e., dispersity (Ð)) values were calculated against the polymethylmethacrylate (PMMA) standards from polymer standard service (PSS). The infrared (IR) spectra were measured on a Perkin-Elmer 1600 series FTIR spectrometer in attenuated total reflectance (ATR) mode and are reported as wavenumbers (cm^−1^). Lyophilization was performed on a Martin Christ freeze dryer (model Alpha 2–4 LSC plus). High-speed vibration milling (HSVM) was performed in a Fritsch Mini-Mill Pulverisette 23 in a 10 mL stainless steel grinding bowl with a grinding ball 15 mm in diameter. Preparative SEC was performed on disposable PD-10 desalting columns from GE Healthcare, following the gravity protocol described in the accompanied instructions. The polymerizations were performed in capped vials in a single mode microwave Biotage initiator sixty (IR temperature sensor).

### 2.3. Synthesis of Poly(2-ethyl-2-oxazoline)_90_-stat-poly(2-C3Mest-2-oxazoline)_10_ Copolymer (Poly(EtOx-stat-C3MestOx))

The copolymer was synthesized in accordance with the literature [58]. EtOx (5.447 mL, 54 mmol), C3MestOx (1.03 mL, 6 mmol), MeOTs (0.090 mL, 0.6 mmol), and acetonitrile (ACN) (8.52 mL) were added to a 20 mL microwave vial and then polymerized in the Biotage microwave for 12 min at 140 °C. Termination of the polymerization was done by the addition of a fourfold molar excess of piperidine relative to the MeOTs. The polymer was isolated by precipitation in a tenfold excess of Et_2_O. After decanting the Et_2_O, the polymer was dissolved in water and freeze dried to obtain a white powder (yield = 92% ^1^H NMR (500 MHz, DMSO-*d*_6_) δ 3.58 (m, 33H), 2.44–2.12 (m, 227H), 1.69 (m, 20H), 1.61–1.30 (m, 10H), 0.97 (m, 270H); SEC: M_n_ = 11.7 kDa, Ð = 1.14).

### 2.4. TBD-Catalyzed Amidation of the Poly(2-ethyl-2-oxazoline)_90_-stat-poly(2-C3Mest-2-oxazoline)_10_ Copolymer with 1-adamantanemethylamine (P(EtOx-stat-AdamantanOx))

The amidation procedure was performed according to a procedure reported earlier [59]. In short, 100 mg of the polymer (1 eq., 0.93 mmol of methyl ester groups), 93 mg of 1-adamantanemethylamine (6 eq., 5.58 mmol), and 39 mg of TBD (3 eq., 2.79 mmol) were added to a stainless steel grinding vessel along with a steel ball 15 mm in diameter. Next, the grinding vessel was mounted onto the HSVM device and agitated for 4 h at a frequency of 50 Hz. Upon completion, 2 mL of water was added in order to redisperse the polymer. This solution was then further diluted and neutralized and subsequently purified by passing the solution over a PD-10 desalting column. Finally, the polymer was isolated in a yield of 82% by freeze drying the aqueous solution (^1^H NMR (400 MHz, DMSO-*d*_6_) δ 7.61 (m, 7H), 3.92–2.88 (m, 536H, H_2_O contamination) 2.41–2.01 (m, 236H), 1.92 (28H), 1.78–1.51 (m, 70H), 1.42 (55H), 1.29–1.19 (m, 6H), 0.96 (270H); SEC: M_n_ = 10.3 kDa, Ð = 1.20)

### 2.5. Cloud Point Measurements

The cloud points of the polymers were determined via parallel turbidimetry, which was performed on either 5 mg/mL or 10 mg/mL polymer solutions in D_2_O using an Avantium Crystal16 parallel crystallizer turbidimeter. The samples were heated and cooled at 1 °C/min while stirring at 700 revolutions per minute. Three heating and cooling cycles were performed. The T_cp_s and clearance points (T_cl_s) were determined as the temperature at which 50% transmittance was reached, and they are reported here as an average of the three heating and cooling cycles with error bars.

### 2.6. ^1^H NMR Titration Experiments

The ^1^H NMR spectroscopy titration experiment was performed on a 400.13 MHz Bruker Avance Il spectrometer. Here, a 5 mg/mL polymer solution was titrated with aliquots of a 100 mg/mL hydroxypropyl-β-CD stock solution, and 32 scans were recorded for each point with a delay time of 2 s. All spectra were referenced using the residual H_2_O solvent signals at 4.79 ppm.

### 2.7. Diffusion Ordered NMR Spectra (2D DOSY)

The ^1^H experiments were performed using the zg pulse program from the standard Bruker library (90° pulse–acquire sequence). For the ^1^H experiments, the spectral width used was 19 ppm with 8 scans of 65,000 data points each being accumulated, preceded by 8 dummy scans. A relaxation delay (d1) of 1 s was used throughout, and the spectrometer excitation frequency (O1) was set to 5.0 ppm. Processing consisted of one order of zero filling to 65,000 real data points, followed by exponential apodization using a 0.30 Hz line broadening factor prior to Fourier transformation, followed by phase correction and a zero-order baseline correction. The spectra were referenced using the residual H_2_O solvent signals at 4.79 ppm. Pulsed field gradient stimulated spin echo (PFGSTE) translational diffusion or DOSY measurements were performed by using a convection-compensated sequence; more specifically, a double stimulated echo with monopolar gradients with an extended phase cycle was used [60,61]. The magnetic field z-gradients were calibrated at 65.6 G·mm^−1^. The diffusion encoding–decoding gradients were varied linearly between 2% and 98% of their maximum output over 16 or 32 increments. The duration of these gradients and the diffusion delay time were chosen so that, at the highest gradient strength, the intensity of the signals of interest was decreased to at least 10% of the intensity at the lowest gradient strength. The obtained intensity decays were fitted using the built-in diffusion processing suite of Topspin 3.X.

## 3. Results and Discussion

### 3.1. Polymer Synthesis

**Figure 2:**
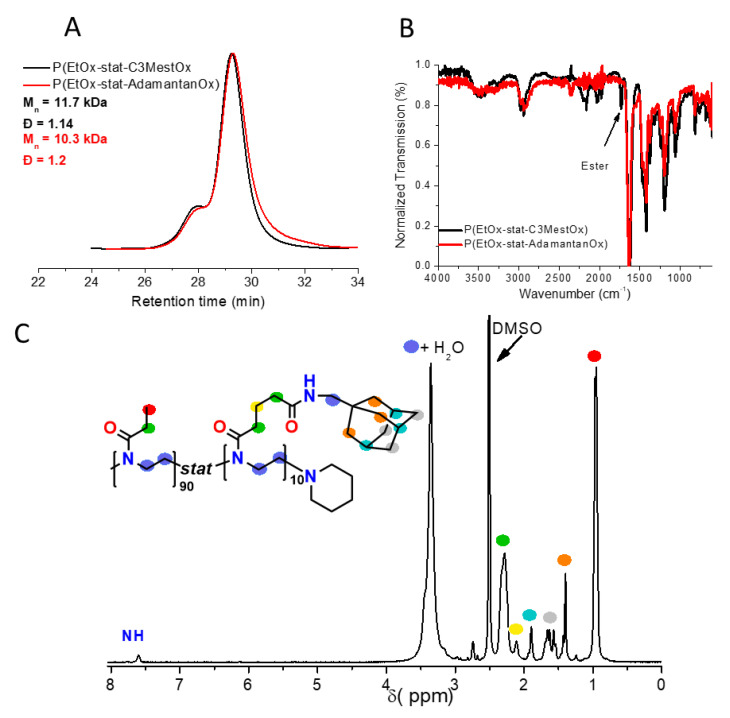
(**A**) Normalized RI traces of P(EtOx-*stat*-C3MestOx) in black and P(EtOx-*stat*-AdamantanOx) in red, with M_n_ and Ð relative to PMMA standards. (**B**) FTIR spectra of P(EtOx-*stat*-C3MestOx) in black and P(EtOx-*stat*-AdamantanOx) in red. (**C**) Annotated ^1^H NMR spectrum of P(EtOx-*stat*-AdamantanOx) measured in DMSO-d6 at room temperature. End-groups were not annotated for clarity reasons.

In order to obtain adamantane functional PAOx, a copolymerization of 2-ethyl-2-oxazoline (EtOx) with 2-methoxycarbonylpropyl-2-oxazoline (C3MestOx) with a feed ratio of 9:1 was performed, where the EtOx provided overall water solubility while the C3MestOx enabled straightforward modification with a wide variety of primary amines [59,62,63]. The statistical copolymerization was initiated with MeOTs under microwave irradiation and was terminated with piperidine. The obtained statistical copolymer poly(EtOx-*stat*-C3MestOx) was well-defined, with a Ð below 1.2, although a minor double molecular weight shoulder can be observed (Figure 2A, black curve), which is a common feature for PAOx prepared at elevated temperatures and in high monomer conversion [64]. In fact, PAOx copolymers often have more pronounced shouldering than their homopolymer counterparts of a similar molecular weight [65]. Finally, the ^1^H NMR spectrum (Figure S1) shows that the polymer composition closely matched the feed ratio. Next, the ester groups of the copolymer were modified with 1-adamantane methyl amine via a triazabicyclodecene (TBD)-catalyzed amidation, using a mechanochemical approach that was environmentally friendly [66], simplified the work-up, and provided a high concentration of reagents to ensure rapid and full conversion of the methyl ester groups. The 1-adamantane methyl amine was chosen over the more sterically demanding—but biologically active—1-adamantane amine [67,68], as substitutions on esters are known to be sterically controlled [69,70]. Figure 2B shows the successful incorporation of the 1-adamantane methyl amine, as the ester signal at 1730 cm^−1^ in the FTIR spectrum disappeared after functionalization. Furthermore, the ^1^H NMR spectrum in Figure 2C shows the broad but characteristic ^1^H signals for adamantane, which confirms their attachment to the polymer, while the observed ratio of adamantane protons to EtOx protons closely matched the ratio of C3MestOx to EtOx protons of the starting material, which is indicative of quantitative conversion (see Figures S1 and S2). In addition, DOSY NMR showed that the observed adamantane protons had a comparable diffusion coefficient to the polymer in D_2_O, which confirms their covalent attachment and confirms the absence of free adamantane (Figure S3). Finally, the refractive index (RI) signal obtained from SEC showed the absence of additional chain coupling reactions, while the well-defined nature of the size-distribution was maintained. However, the polymer did not show a clear shift in retention time, which can be attributed to the strong hydrophobicity of the adamantane group, which reduced the hydrodynamic volume of the polymer in the DMA mobile phase, despite the increase in the absolute molecular weight of the polymer. This, therefore, led to an observed decrease in the relative molecular weight versus the PMMA standards upon modification. This reduction in polarity was also observed upon assessing the T_cp_ of the P(EtOx-stat-AdamantanOx) at 5 mg/mL, which was 32 °C, whereas the T_cp_ of the starting material was reported to be 89 °C [58].

### 3.2. Tuning Thermoresponsive Behavior by Molecular Recognition

Next, the thermoresponsive behavior of the polymer was explored by turbidimetry in the presence of β-CD or hydroxypropyl-β-CD (HP-β-CD). Figure 2 shows the results obtained for a 10 mg/mL and a 5 mg/mL polymer solution with increasing β-CD and HP-β-CD contents, respectively. It should be noted that, due to the low water solubility of β-CD (18.5 mg/mL), the use of a stock solution was not possible, as this would entail significant dilution of the polymer solution (i.e., for every 0.2 eq, approximately 0.1 mL would have to be added), which would significantly affect the obtained values. Hence, for each titration step, the β-CD was weighed, and the polymer solution was subsequently added to the solid host and measured. To minimize any practical weighing errors, which might be more pronounced at lower concentrations 10 mg/mL was chosen, although the data obtained at 5 mg/mL corresponded well to that obtained at 10 mg/mL (Figure S4).

**Figure 3:**
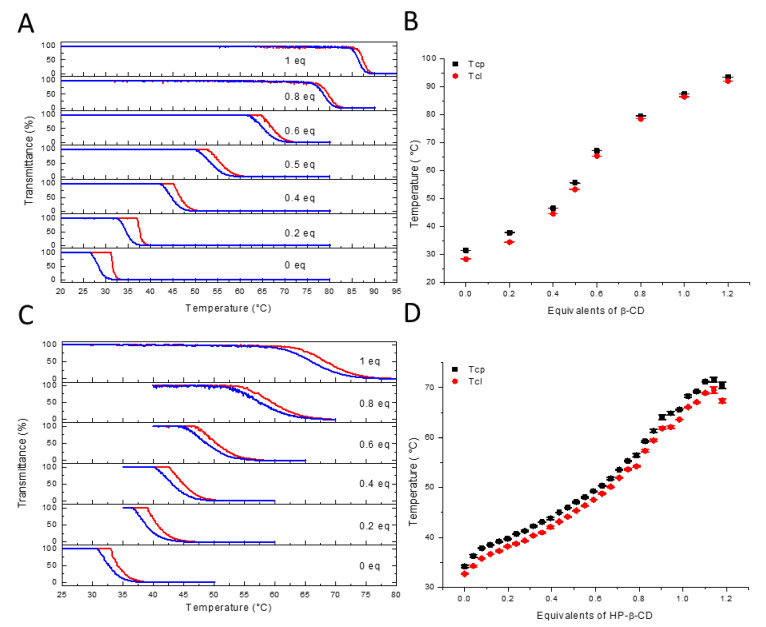
(**A**) Stacked turbidimetry plots of a 10 mg/mL solution of P(EtOx-stat-AdamantanOx) with a varying β-CD content, showing heating and cooling curves in red and blue, respectively. (**B**) Observed T_cp_ and T_cl_ as a function of the β-CD content for a 10 mg/mL solution of P(EtOx-stat-AdamantanOx) with error bars (n = 3). (**C**) Stacked turbidimetry plots of a 5 mg/mL solution of P(EtOx-stat-AdamantanOx) with a varying HP-β-CD content. (**D**) Observed T_cp_ and T_cl_ as a function of HP-β-CD content for a 5 mg/mL solution of P(EtOx-stat-AdamantanOx) with error bars (n = 3).

Figure 3A,C shows the heating and cooling curves of the second heating run in the presence of a 0–1 equivalent of β-CD and HP-β-CD, respectively. Overall, the thermal transitions are sharp and display minimal hysteresis (<3.4 °C) between the cooling and heating curves. This is in contrast with our earlier work, where a hysteresis of 10 °C (at the same heating rate) was observed for CD complexes with a poly(2-ethyl-2-oxazoline-*ran*-2-nonyl-2-oxazoline) random copolymer, which was attributed to partial decomplexation upon heating [40]. The lack of significant hysteresis in this work suggests that the complexation of the adamantine side chains with CD was thermodynamically favorable over the investigated temperature range (i.e., complex formation does not impose a significant entropic penalty), and that the polymer collapse occurred with intact host–guest complexes (i.e., the CD was entrapped in the collapsed polymer phase). This can be partially attributed to the relatively high association constants (K_a_s) of the adamantane β-CD complexes, typically in the order of 10^4^ [71,72,73,74,75,76], which is a hundredfold higher than the K_a_s reported for the poly(2-ethyl-2-oxazoline-*ran*-2-nonyl-2-oxazoline) random copolymers [40]. For 1-adamantanemethylamide derivatives in particular, a relatively high K_a_ with β-CD has been reported, viz. 5.2 × 10^4^ [77].

**Figure 4:**
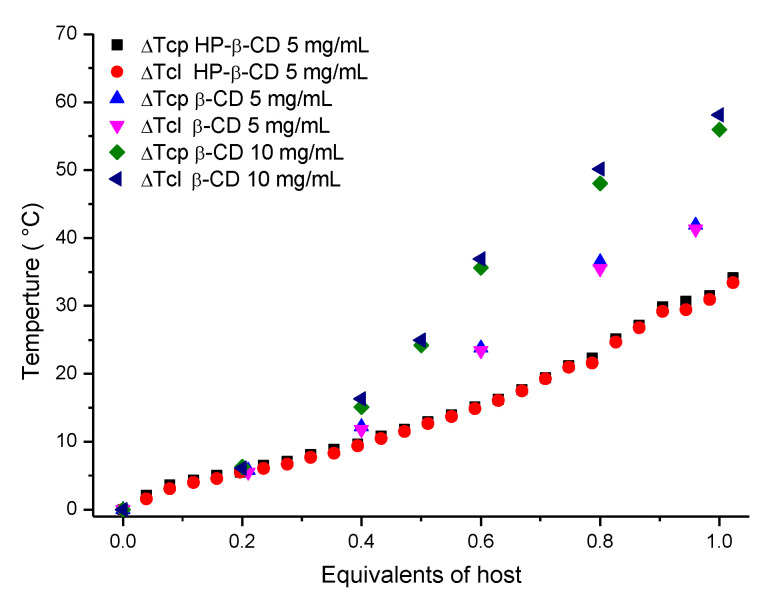
∆T_cp_ of P(EtOx-stat-AdamantanOx), plotted as a function of the host content.

Next, the average T_cp_s and T_cl_s obtained from three heating and cooling runs were plotted as a function of the equivalents of the β-CD and HP-β-CD added (Figure 3B,D, respectively). Note that above 1.2 equivalents of β-CD, no T_cp_ could be detected, as the mixture remained transparent over the entire temperature range. This observation suggests that the inclusion complex was relatively polar, as poly(2-ethyl-2-oxazoline) of a similar chain length has a T_cp_ of ± 91 °C under these experimental conditions [33]. Furthermore, this indicated that not all adamantane groups are fully complexed at a 1.2 ratio of CD:adamantane. Plotting the T_cp_ and T_cl_ as a function of the equivalents of the host added should allow the determination of the association constant in a similar fashion to our previous work [40] when a 1:1 binding stoichiometry is assumed. Rather than obtaining typical binding isotherms for 1:1 complexation, viz. hyperbolic functions, more complex sigmoidal relationships were obtained, which were indicative of positive cooperativity. It should be noted that the turbidimetry measurements probed both the inclusion complex formation and temperature-induced phase separation, and that the observed positive cooperativity could be related to both phenomena. While it cannot be excluded, it seems unlikely that the binding of one β-CD to the multivalent polymer would lower the energetic barriers for subsequent binding events and thereby facilitate positive cooperativity, as was also confirmed by the isothermal determination of the K_a_ by ^1^H NMR spectroscopy, which did not indicate cooperativity, vide infra. A more likely explanation is that the cooperativity was related to the temperature-induced phase separation, where it may be speculated that subsequent binding events prevented the formation of intermolecular hydrophobic adamantane clusters, thereby leading to a cooperative enhancement of water solubility and not just a change in the hydrophilic–hydrophobic balance. In addition, the presence of multiple CD host–guest complexes along the polymer chain may have facilitated cooperative hydrogen bonding between the polymer and the solute, further promoting water solubility. Due to the existence of this large variety of possible secondary interactions, the direct determination of the association constant from correlation of the T_cp_ with the equivalents of CD was not possible, which demonstrates the limitations of T_cp_ as a physical parameter for the determination of association constants. Nonetheless, the variation in T_cp_ as a function of the host content can still be useful to compare the complexes formed with different hosts. Figure 4 shows that, initially, both hosts elicited similar responses, which could be related to the overall reduction of hydrophobicity due to shielding of the adamantane groups, though the collapse of the polymer chains was still mainly driven by the hydrophobic interactions of the adamantane groups. Beyond 0.4 equivalents, rather big differences in ∆T_cp_ occurred between the different hosts, where β-CD elicited larger shifts. The smaller shifts elicited by HP-β-CD were likely associated with its larger hydrophobic cavity, which resulted in the partial shielding or dehydration of the polar secondary amide group. The shielding and dehydration of the secondary amide reduced the cooperativity due to reduced hydrogen bonding, therefore resulting in a net ∆T_cp_ of ± 30 °C for the HP-β-CD:polymer inclusion complexes at equimolarity. For the β-CD, shielding of the secondary amide should be minimal; therefore a larger ∆T_cp_ of ± 42 °C was observed at equimolarity for a 5 mg/mL solution, which increased to 56 °C for a 10 mg/mL solution, indicating a larger extent of complexation at a higher concentration. Presumably, the operating window could be increased further by optimizing the adamantane content in the polymer or polymer concentration, thus presenting a viable alternative to exhaustive copolymer synthesis for providing a thermal response at any given temperature.

### 3.3. Molecular Recognition of Adamantane Pendant Groups by Hydroxypropyl-β-CD

**Figure 5:**
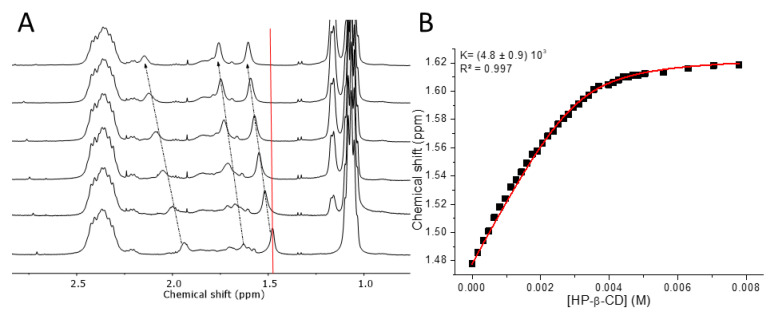
(**A**) Stacked ^1^HNMR spectra of the titration of P(EtOx-*stat*-AdamantanOx) (5 mg/mL in D_2_O with an increasing HP-β-CD concentration in increments of 0.2 equivalents, relative to the adamantane groups from bottom to top). The arrows and red line indicate the peaks of interest and guide the eye. (**B**) Binding isotherm obtained from the titration experiment, whereby the chemical shift is plotted as a function of the HP-β-CD concentration. The applied fitting is displayed in red.

Next, the molecular recognition between P(EtOx-*stat*-AdamantanOx) and hydroxypropyl-β-CD (HP- β-CD) was investigated via ^1^H NMR spectroscopy titration in D_2_O. HP-β-CD was chosen instead of β-CD due to its higher water solubility (i.e., up to 50% w/v in water compared with 18.5 g/L for β-CD), therefore leading to minimal dilution of the polymer solution over the course of the titration experiment. Figure 5A shows the downfield shifting of the original adamantane signals (δ_Ad_) over the course of the titration experiment, which is indicative of inclusion complex formation with HP-β-CD, whereby upon full complexation, δ_CD-Ad_ would be reached (full spectra are provided in Figure S5). The gradual downfield shifting of the adamantane signals also suggest a fast exchange between complexed and non-complexed species on the NMR time scale. This is in line with the turbidimetry experiments and literature, where a fast exchange was observed between β-CD and adamantane functional polymers with long spacers between the pendant adamantane groups and the polymer backbone [45].

Subsequent plotting of the chemical shift (δ_obsd_) of the adamantane signal around 1.48 ppm as a function of the HP-β-CD concentration ([CD]_0_) enabled the determination of the association constant (K) between the pendant adamantane groups and the HP-β-CD via non-linear regression of the binding isotherm. This signal was chosen as it remained clearly resolved throughout the titration and did not overlap with signals from the host or the polymer terminus. In contrast to the data obtained from turbidimetry, the binding isotherm had the expected hyperbolic shape, therefore confirming that the positive cooperativity observed earlier was associated to the phase separation event itself and not to the host–guest binding. Furthermore, the binding isotherm suggests that HP-β-CD binds in a 1:1 fashion to the pendant adamantane groups on the polymer. Therefore, the binding constant can be determined via well-established non-linear regression analysis [78,79,80], whereby the binding isotherm is fitted to the following equation [81,82]:

δ_obsd_ = δ_Ad_ + (1 − [Ad]/[Ad]_0_)(δ_CD-Ad_ − δ_Ad_)

This equation is fitted with the following:

[Ad] = {K[Ad]_0_ − K[CD]_0_ − 1 + √((K[Ad]_0_ + K[CD]_0_ + 1)^2^ − 4K^2^ [Ad]_0_[CD]_0_)}/2K

The obtained K_a_ of 4.8 × 10^3^ was an order of magnitude lower than what is typically described for 1-adamantanemethylamide derivatives in particular, viz. 5.2 × 10^4^ [77]. However, the observed order of magnitude lower K_a_, compared to native β-CD complexes, is not uncommon for inclusion complexes formed with HP-β-CD, where the observed association constants drop as the hydroxylpropylation degree increases [83]. This drop has been attributed to their extended hydrophobic cavity, which imposes an enthalpic penalty due to dehydration of the polar groups on the guest. The complexation of the pendant adamantane groups with HP-β-CD was therefore driven by contributions of both enthalpy and entropy, whereas complexation with native β-CD was mainly enthalpy driven [84]. It has been described that, in the case of neutral polar groups, in this case a secondary amide, the net gain in the entropic factor is unable to compensate for the increased enthalpy, leading to a tenfold decrease in the association constant [83]. These results further support the observations made from the turbidimetry measurements and highlight the importance of linker and host compatibility in the synthesis of thermoresponsive supramolecular assemblies.

### 3.4. Diffusion Ordered NMR Spectroscopy

**Figure 6:**
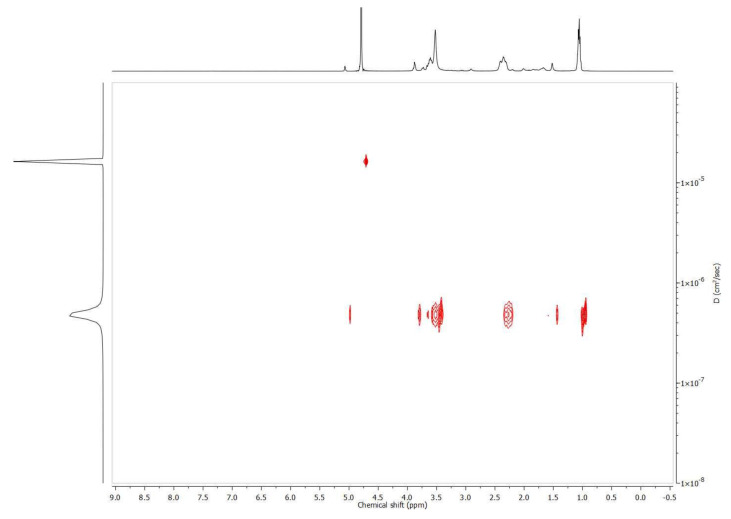
Two-dimensional diffusion ordered spectroscopy (2D DOSY) NMR spectrum of P(EtOx-*stat*-AdamantanOx) with 0.2 equivalents of β-CD at 25 °C in D_2_O.

Next, the single-chain behavior of the polymers and the polymer β-CD inclusion complexes was studied as a function of temperature by DOSY NMR spectroscopy (Figures S6–S9). From the DOSY measurements, the hydrodynamic radius could be calculated via the obtained diffusion constant and the Stokes–Einstein equation, assuming a spherical shape for all components. For the calculation of the hydrodynamic radius, the temperature dependent viscosity of D_2_O was taken into account [85]. The results listed in Table 1 show that P(EtOx-stat-AdamantanOx) underwent a small decrease in size from 3.68 nm below the T_cp_ to 3.52 nm above the T_cp_, which corresponds to the collapse of the polymer chains. When β-CD was added to the P(EtOx-*stat*-AdamantanOx), the solvated structure increased in size from 3.68 nm to 4.26 nm, which was indicative of complex formation and improved hydration below the T_cp_. The DOSY spectrum in Figure 6 also shows that the β-CD diffused together with the polymer, further proving the inclusion complex formation. When the inclusion complex was heated above the T_cp_, a strong reduction in size was observed from 4.26 nm to 0.66 nm, indicating that the collapsed polymer globules were no longer observed by DOSY NMR, and only a minor fraction of the released β-CD was observed.

### 3.5. Rationalization of Polymer Design with Respect to Thermal Response

**Figure 7:**
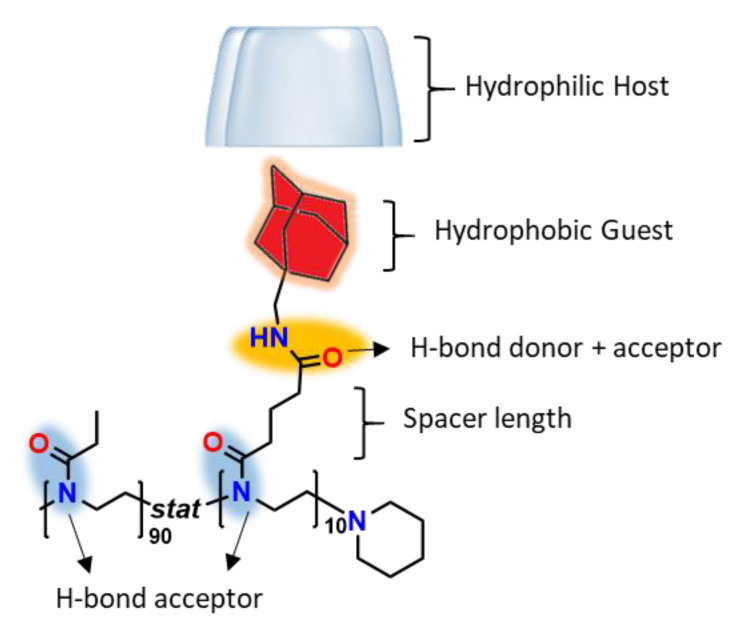
Rationalization of the polymer design.

The obtained results indicate that the observed thermal transitions were the result of the polymer design as a whole and not just the host–guest complexation. Therefore, it can be rationalized that different parameters in the polymer design affect the subtle interplay of supramolecular interactions involved in the temperature-induced phase transition, which will be briefly summarized here and are illustrated in Figure 7. The PAOx backbone provides the necessary hydrophilic–hydrophobic balance for LCST behavior, whereby the hydrophilicity of the H-bond accepting tertiary amide is counteracted by the length of its hydrophobic alkyl substituents. Therefore, the comonomer should be chosen as a function of the relative hydrophobicity of the guest molecule and the number of guest molecules per polymer chain. Here, a secondary amide is installed in the linker between the polymer backbone and hydrophobic guest molecules to enable additional H-bonding and to enhance the overall hydrophilicity, leading to an adamantane functionalized copolymer with a T_cp_ of 32 °C. The spacer length between the polymer backbone can potentially be tuned to vary the exchange rate of the host and guest and therefore plays a crucial part in the occurrence of hysteresis between heating and cooling curves. The host–guest pair (i.e., the association constant and its thermodynamic parameters, as well as the relative dimensions of the host) will also affect the thermal response and its dependence on the host concentration. Finally, the observed cooperativity is hypothesized to be related to the hydrophobic shielding of the adamantane groups and, hence, would depend on the comonomer ratio and polymer chain length.

## 4. Conclusions

The synthesis of polymers with an adaptable thermal response is an attractive alternative to exhaustive copolymer synthesis, requiring limited synthetic effort. In this context, supramolecular complexation is a promising tool to synthesize polymers with a tunable thermoresponsive behavior, yet the application thereof is limited, as most reports require a substantial excess of the host and show a limited window of tunability, viz. 5–20 °C. Within this work, we demonstrate that this apparent limitation can be overcome by combining supramolecular complexation with rational polymer design. In summary, a thermoresponsive poly(2-alkyl-2-oxazoline) copolymer with pendant adamantane groups was prepared in a single step via an organocatalyzed post-polymerization amidation reaction. The synthesized copolymer was capable of forming polymer inclusion complexes via molecular recognition with both β-CD and HP-β-CD. This molecular recognition was subsequently exploited to tune the thermoresponsive behavior of the polymer in the exceptionally wide temperature range of 56 °C by simply adding up to 1 equivalent of β-CD. The observed thermal transitions were sharp and revealed minimal hysteresis over the entire temperature range. This exceptional tunability could be attributed to the subtle interplay of hydrophobic interactions, host–guest recognition, and cooperative hydrogen bonding, which were rationalized in the polymer design as a whole. Together, these results demonstrate that molecular recognition and rational polymer design can be powerful tools in the synthesis of polymers featuring tunable thermal responsiveness with minimal synthetic effort. Future studies will be aimed at the introduction of additional responsiveness in the polymer structure and the exploitation of molecular recognition and thermoresponsive behavior in drug delivery [86,87]. Furthermore, careful exploitation of the polymer structure and host–guest chemistry might enable the synthesis of polymer inclusion complexes with complex thermoresponsive behaviors.

## Figures and Tables

**Table 1:** Diffusion constant and size of P(EtOx-stat-AdamantanOx), β-CD, and their complexes at a 5:1 guest-to-host ratio above and below the T_cp_.

Compound	Df (m^2^/s)	R_h_ (nm)
P(EtOx-stat-AdamantanOx) (25 °C)	5.41 × 10^−11^	3.68
P(EtOx-stat-AdamantanOx) (42 °C)	8.34 × 10^−11^	3.52
β-CD (25 °C)	2.66 × 10^−10^	0.75
β-CD (42 °C)	3.83 × 10^−10^	0.77
Complex (25 °C)	4.68 × 10^−11^	4.26
Complex (42 °C)	4.42 × 10^−10^	0.66

## Data Availability

The data presented in this study are available on request from the corresponding author.

## Supplementary Materials

The following are available online at https://www.mdpi.com/2073-4360/13/3/374/s1: additional ^1^H NMR and DOSY NMR spectra.
