# Supplementary material for: Adamantane Functionalized Poly(2-oxazoline)s with Broadly Tunable LCST-Behavior by Molecular Recognition
Source: Polymers (Basel). 2021 Jan 26;13(3):374. doi: 10.3390/polym13030374 (PMC7865518; doi:10.3390/polym13030374)
Supplement: Supplementary file 1 [file polymers-13-00374-s001.pdf]

Supporting Information:

Adamantane functionalized Poly(2-oxazoline)s with broadly tunable LCST-behavior by molecular recognition

Joachim F. R. Van Guyse,[1] Debaditya Bera[1] and Richard Hoogenboom[1,*]

[1] Supramolecular Chemistry Group, Centre of Macromolecular Chemistry (CMaC), Department of Organic and Macromolecular Chemistry, Ghent University, Krijgslaan 281-S4, B-9000 Ghent, Belgium

* Correspondence: Richard.hoogenboom@ugent.be

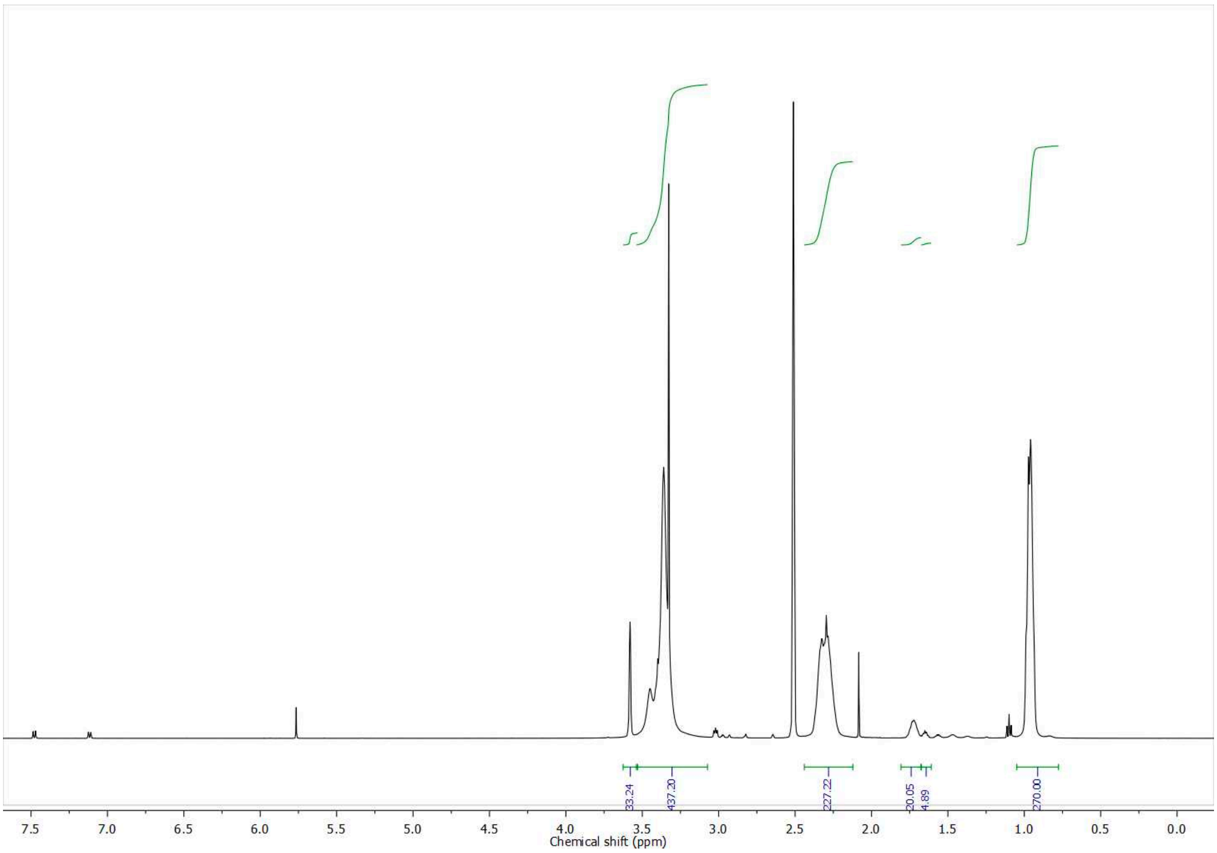

Figure S1. ^{1}H NMR spectrum of P(EtOx-*stat*-C3MestOx) in dmso-d6, with slight water contamination present in the signal at 3.4 ppm.

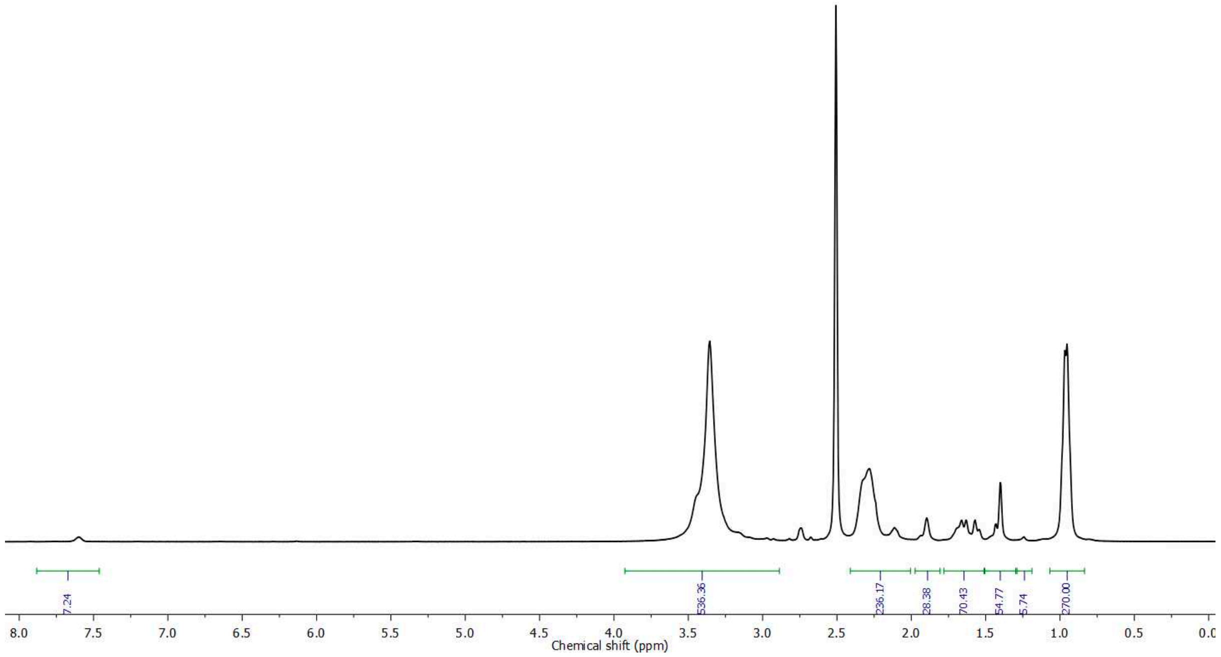

Figure S2. ^{1}H NMR spectrum of P(EtOx-*stat*-AdamantanOx) in dmso-d6, with slight water contamination present in the signal at 3.4 ppm.

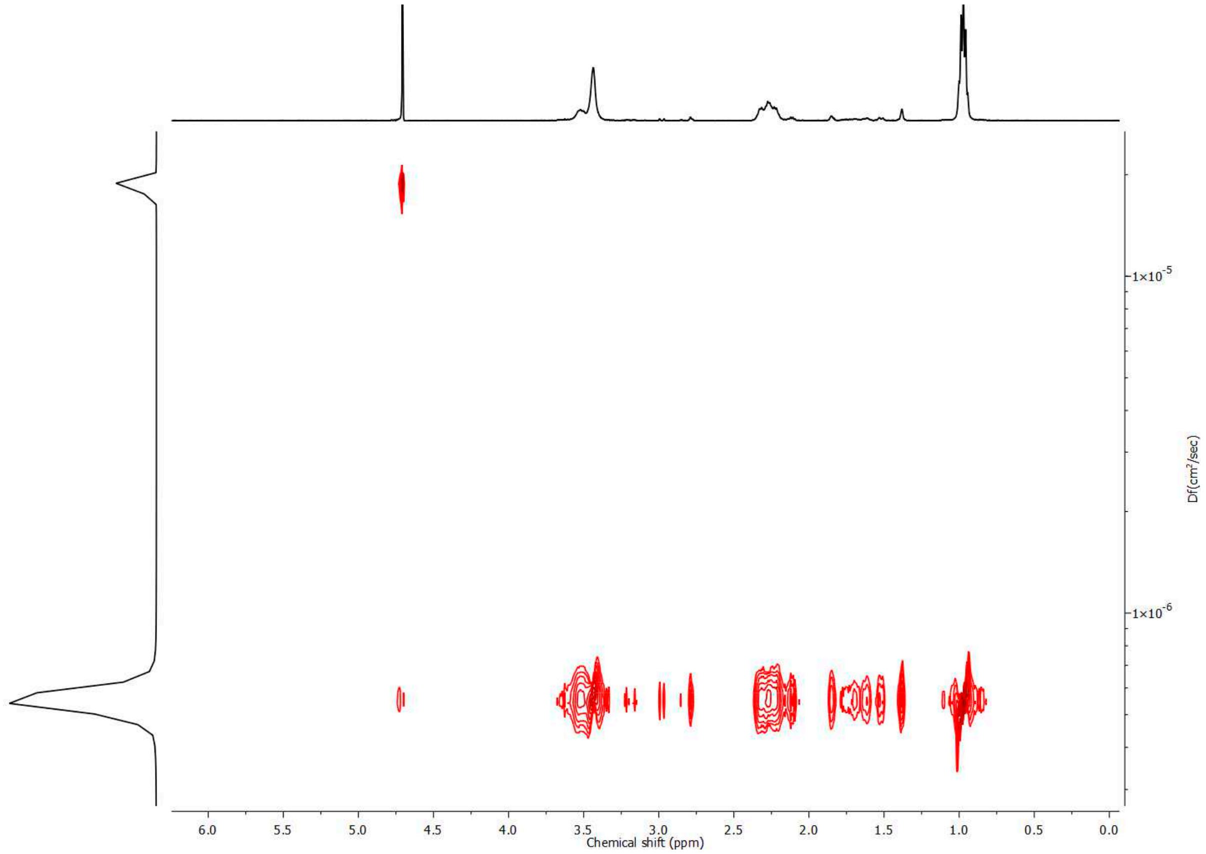

Figure S3. 2D DOSY NMR spectrum of P(EtOx-*stat*-AdamantanOx) in D_2O at 25°C.

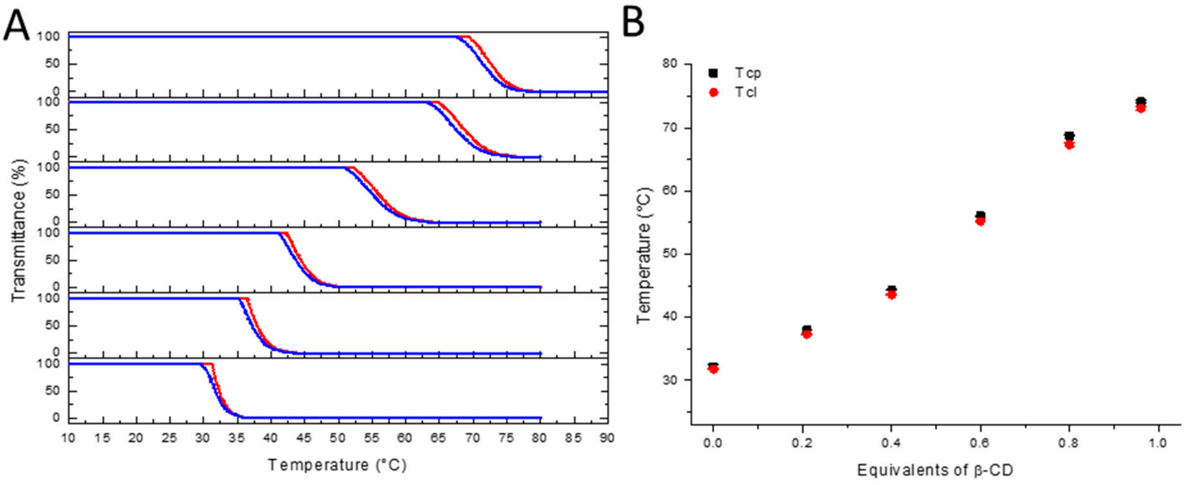

Figure S4.A) Stacked turbidimetry plots of a 5 mg/mL solution of P(EtOx-*stat*-AdamantanOx) with increasing β-CD content from bottom to top. B) Observed T_{cp} and T_{cl} as a function of β-CD content for a 5 mg/mL solution of P(EtOx-*stat*-AdamantanOx) with error bars.

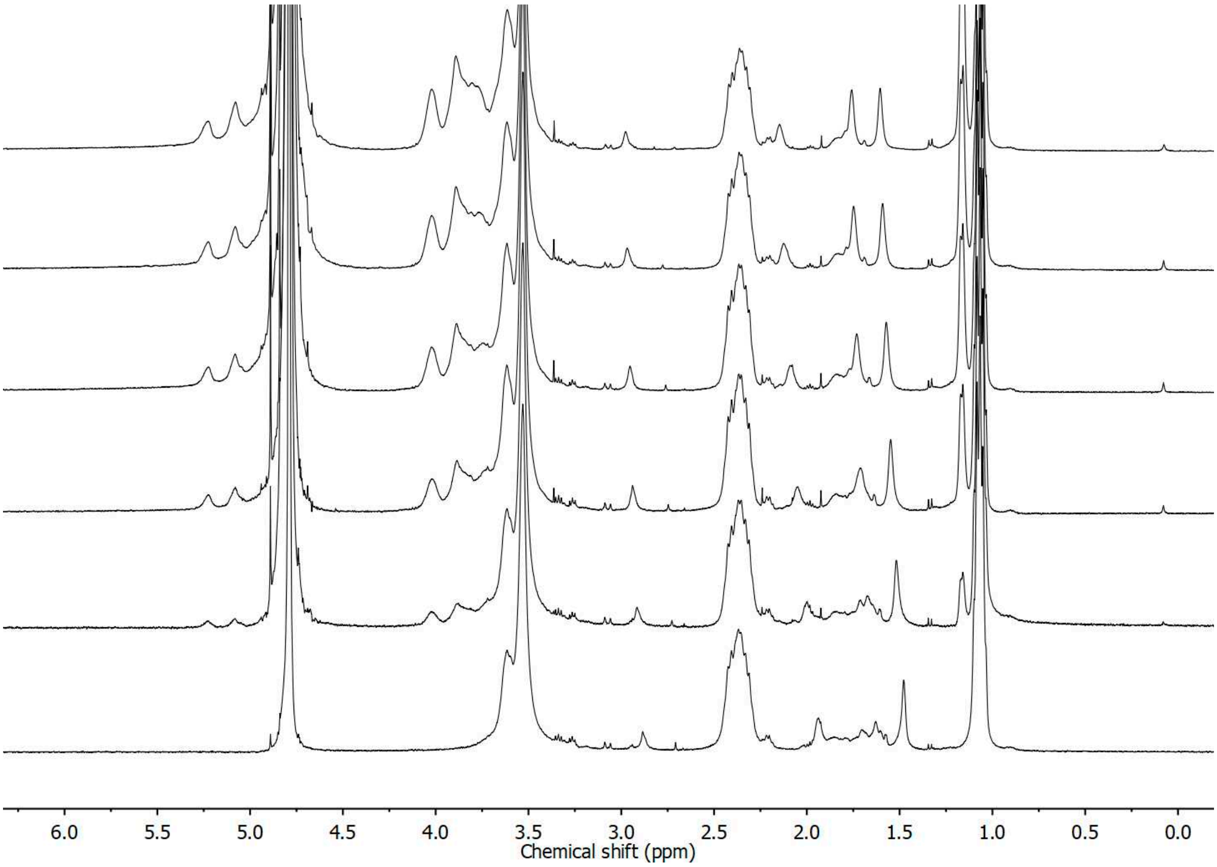

Figure S5. Stacked ^{1}H NMR spectra of P(EtOx-*stat*-AdamantanOx) with increasing HP-β-CD content from bottom to top (0-1 equivalents) in increments of 0.2 equivalents in D_2O .

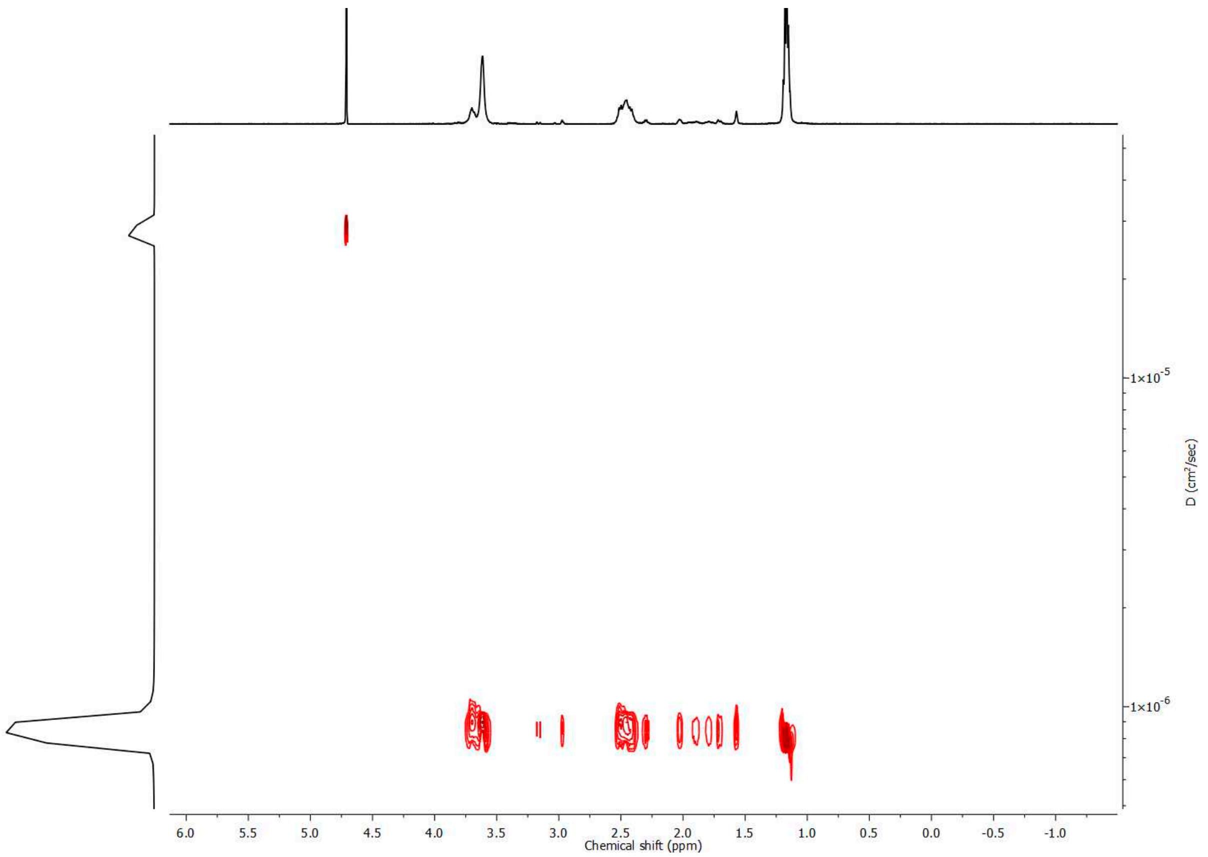

Figure S6. 2D DOSY NMR spectrum of P(EtOx-*stat*-AdamantanOx) in D_2O at 42°C.

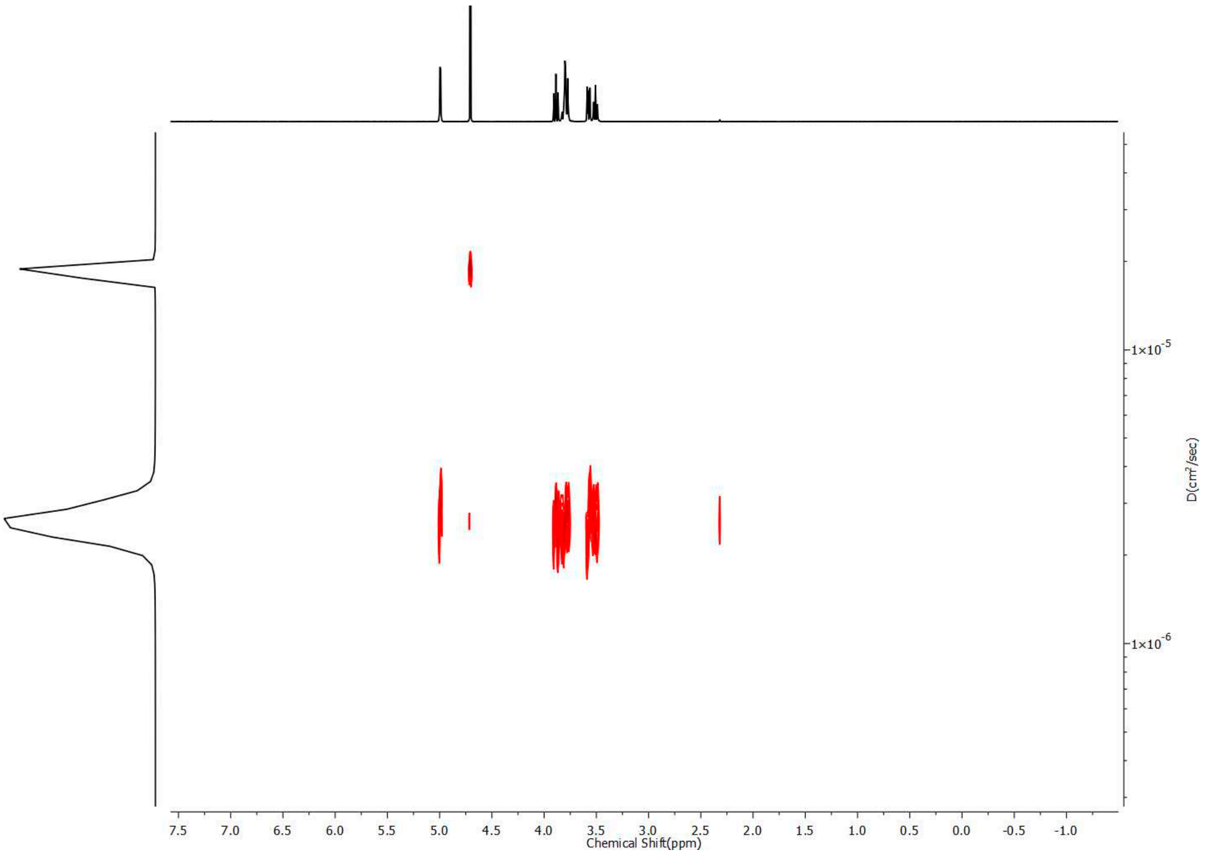

Figure S7. 2D DOSY NMR spectrum of β-CD in D_2O at 25°C.

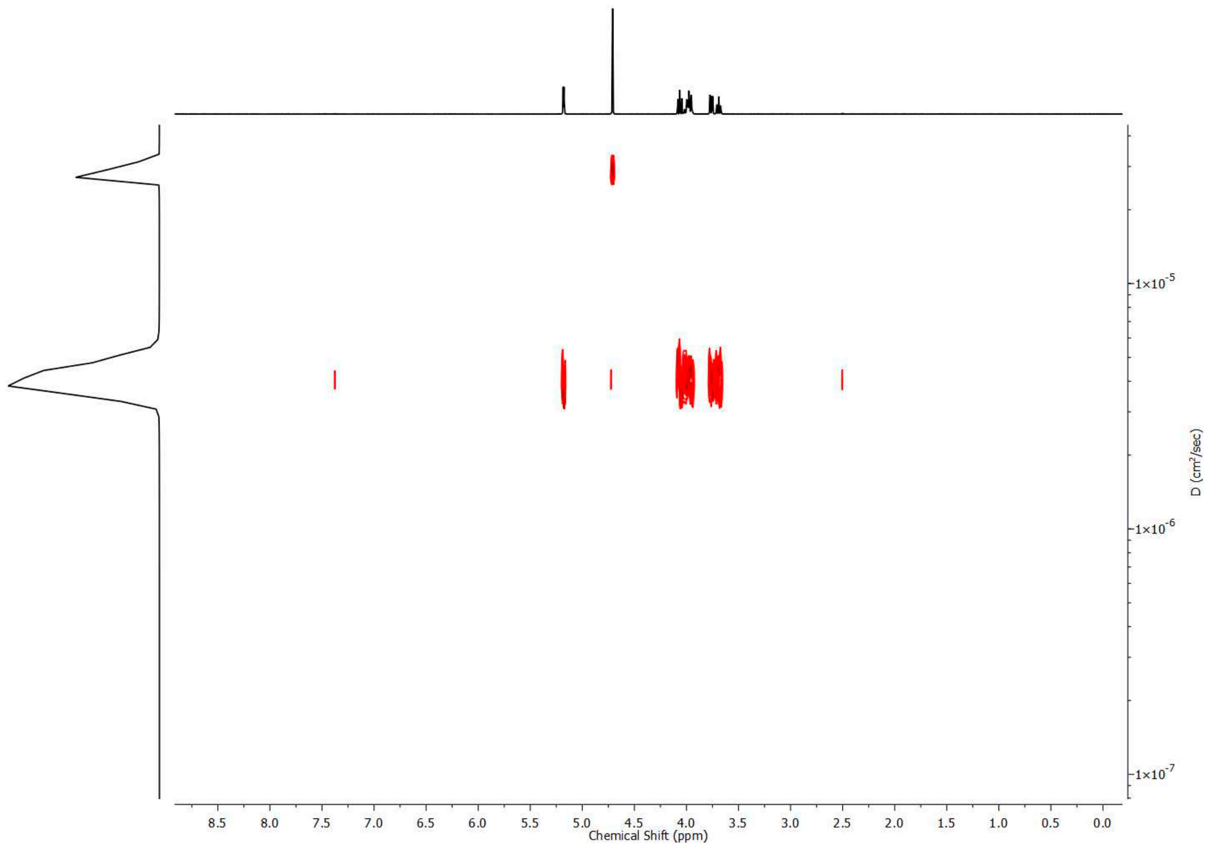

Figure S8. 2D DOSY NMR spectrum of HP-β-CD in D_2O at 42°C.

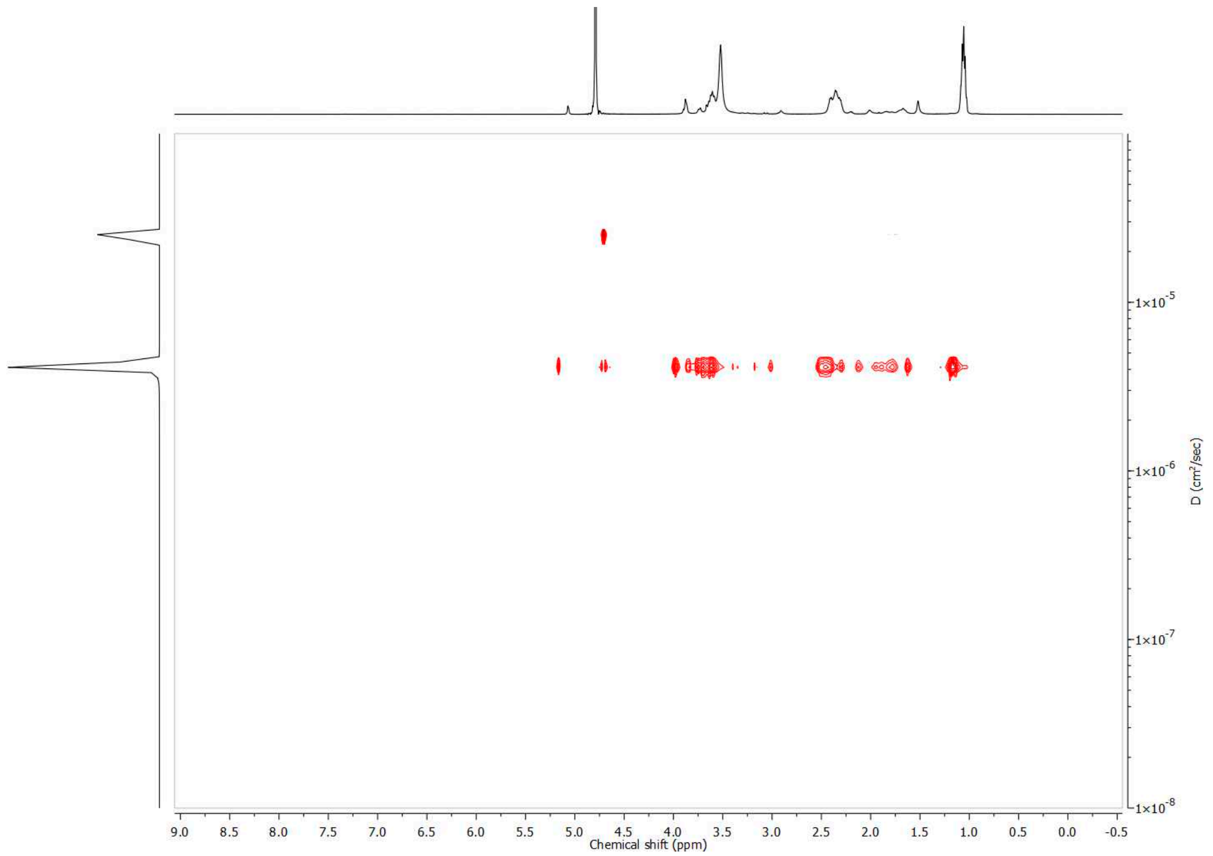

Figure S9. 2D DOSY NMR spectrum of P(EtOx-*stat*-AdamantanOx) with 0.2 eq β-CD in D_2O at 42°C.
